# Controlled Ovarian Stimulation After Fertility-Sparing Surgery for Borderline Ovarian Tumors: An Exploratory Cohort Study on Recurrence and Reproductive Outcomes

**DOI:** 10.3390/curroncol33040206

**Published:** 2026-04-03

**Authors:** Sofia Thiella, Giacomo Corrado, Paola Villa, Inge Peters, Diana Giannarelli, Rossella Letizia Mancusi, Tina Pasciuto, Lucrezia Massaro, Maria Luisa Di Pietro, Maria Lucia Specchia, Anna Fagotti

**Affiliations:** 1 Centre for Research and Studies on Reproductive Health, Catholic University of the Sacred Heart, 00168 Rome, Italy; sofia.thiella@studenti.univr.it (S.T.); marialuisa.dipietro@unicatt.it (M.L.D.P.); 2 Gynaecologic Oncology Unit, Department of Woman’s and Child Health and Public Health Sciences, Fondazione Policlinico Universitario Agostino Gemelli IRCCS, 00167 Rome, Italy; 3 Department of Life Sciences and Public Health Section of Hygiene, Fondazione Policlinico Universitario Agostino Gemelli IRCCS, 00167 Rome, Italy; 4 Department of Woman’s and Child Health and Public Health Sciences, Quality of Life, Integrated Therapies and Oncology Follow-Up, Fondazione Policlinico Universitario Agostino Gemelli IRCSS, 00167 Rome, Italy; 5 Epidemiology and Biostatistics Research Core Facility Gemelli Science and Technology Park (G-STeP), Fondazione Policlinico Universitario Agostino Gemelli IRCCS, 00167 Rome, Italy; 6 Data Collection Research Core Facility Gemelli Science and Technology Park (G-SteP), Fondazione Policlinico Universitario Agostino Gemelli IRCCS, 00167 Rome, Italy; 7 Faculty of Medicine and Surgery, Catholic University of the Sacred Heart, 00168 Rome, Italy

**Keywords:** borderline ovarian tumor, fertility-sparing surgery, controlled ovarian stimulation, oocyte cryopreservation, pregnancy

## Abstract

Borderline ovarian tumors often affect women of reproductive age for whom fertility preservation may be a major concern. While fertility-sparing surgery is widely accepted, the safety of controlled ovarian stimulation for oocyte cryopreservation after surgery remains uncertain due to potential recurrence risk. This retrospective single-center cohort study explored the association between controlled ovarian stimulation following fertility-sparing surgery and both recurrence risk and reproductive outcomes. Patients treated between January 2011 and June 2024 were included. Baseline characteristics, surgical details, and reproductive outcomes were compared using non-parametric and exact tests. Progression-free survival was assessed with Kaplan–Meier estimates and Cox proportional hazards models, modeling stimulation as a time-dependent covariate, and using a 10-month landmark analysis to account for immortal time bias. Of 45 included patients, 19 underwent ovarian stimulation after surgery, with a median interval of 9.9 months to stimulation. Median follow-up was 34.4 months. Recurrence occurred in 21.1% of stimulated patients versus 38.5% of non-stimulated patients (*p* = 0.338). After adjustment for time-dependent exposure, ovarian stimulation was not associated with increased recurrence risk (HR 0.95, 95% CI 0.22–4.09; *p* = 0.944). Eleven patients (24.4%) achieved at least one pregnancy, 81.8% of which were spontaneous. Controlled ovarian stimulation for oocyte cryopreservation after fertility-sparing surgery for borderline ovarian tumors did not show an increased recurrence risk, although larger studies are needed.

## Simple Summary

Women with borderline ovarian tumors are often young and wish to preserve their fertility. Fertility-sparing surgery allows the uterus and part of the ovary to be maintained, but concerns remain about whether ovarian stimulation to freeze eggs after surgery could increase the risk of tumor recurrence. This study evaluated women who underwent fertility-sparing surgery for borderline ovarian tumors, comparing those who later had ovarian stimulation for egg freezing with those who did not. Over a median follow-up of nearly three years, ovarian stimulation was not associated with a higher risk of tumor recurrence. Importantly, almost one quarter of women achieved at least one pregnancy, mostly without assisted reproductive techniques. These findings suggest that ovarian stimulation for fertility preservation after fertility-sparing surgery may be a feasible option for women with borderline ovarian tumors. Larger studies are needed to confirm these results and to guide clinical decision-making and counseling in oncofertility care.

## 1. Introduction

Borderline ovarian tumors (BOT) are epithelial tumors of uncertain malignant potential without massive stromal invasion at histological examination [1]. They account for approximately 15–20% of all ovarian epithelial tumors [2], with a reported incidence ranging from 1.8 to 4.8 per 100,000 females per year [3]. Given the occurrence of BOT in women of childbearing age, fertility-sparing surgery (FSS) is considered the gold standard treatment according to the recent ESGO-ESHRE-ESGE guidelines [4], except in cases involving invasive peritoneal implants. The FSS technique is based on the preservation of the uterus and at least one part of one ovary. However, FSS is associated with a notable recurrence risk, reported in the literature to range from 7% to 21% [5,6,7]. After FSS, the spontaneous pregnancy rate is highly variable (56–81%) [8,9,10]. Among the fertility-sparing approaches, cystectomy is associated with better fertility results compared to salpingo-oophorectomy (level of evidence IV, grade B) [4].

According to the ESGO-ESHRE-ESGE guidelines [4], patients desiring future fertility should receive reproductive counseling prior to any oncological treatment. When fertility preservation is considered, controlled ovarian stimulation (COS) followed by oocyte cryopreservation (OS) may be performed, with a recommendation level of evidence V, grade D [4]. While numerous studies in the literature have investigated the risk of developing BOT associated with COS [11,12,13], only a limited number of studies have investigated the risk of recurrence in BOT patients undergoing COS for fertility treatment after FSS [14,15,16].

Therefore, the primary objective of this exploratory analysis was to assess whether COS following FSS for BOT is associated with signals of increased recurrence risk compared with FSS alone. Secondary objectives were to describe progression-free survival (PFS) and reproductive outcomes following treatment.

## 2. Materials and Methods

This retrospective, single-center observational cohort study was conducted in accordance with STROBE guidelines [17].

The cohort included women who underwent FSS for BOT as first-line treatment at the Fondazione Policlinico Universitario (FPG) A. Gemelli—IRCCS—Rome, Italy, between January 2011 and June 2024. Subsequent COS and OC, when performed, occurred at non-affiliated institutions. Inclusion criteria were age 18–40 at BOT diagnosis, serous, mucinous, endometrioid, clear cell, or mixed serous–mucinous histology, and a minimum follow-up of one year after FSS. Exclusion criteria included stromal microinvasion > 5 mm^2^ and the diagnosis of another malignancy within five years of the initial BOT surgery.

All data were checked for plausibility and completeness by two authors (S.T., G.C.). Comprehensive data were collected for each patient, encompassing demographic and lifestyle variables such as family status, smoking habits, and body mass index (BMI). Medical history included current therapies, prior surgeries unrelated to BOT, and familial predispositions. Obstetric and reproductive data were documented, including gravidity, parity, and fertility preservation strategies. All included patients presented to FPG with the explicit intention of attempting conception, as self-reported during clinical consultation.

The denominator used to estimate pregnancy rates included all patients who actively attempted to conceive during the study period, defined as those exposed to the risk of conception either through natural intercourse or assisted reproductive techniques. A pregnancy attempt was defined as a continuous period of intentional exposure to conception risk, characterized by regular unprotected intercourse without contraceptive use. In patients undergoing in vitro fertilization (IVF), a pregnancy attempt was defined as a single initiated treatment cycle aimed at achieving pregnancy, including controlled ovarian stimulation, oocyte retrieval, and embryo transfer or planned transfer.

Disease-specific information comprised age at first diagnosis, date of the initial surgical procedure, type of fertility-sparing surgery (cystectomy, unilateral oophorectomy, unilateral salpingo-oophorectomy, unilateral salpingo-oophorectomy + contralateral cystectomy) and tumor characteristics such as laterality, isotype (serous, mucinous, endometrioid, clear cell, mixed), presence of micropapillary features, and implant status. For patients experiencing recurrence, details regarding subsequent surgical procedures, tumor laterality at recurrence, time to recurrence, and the presence of implants were recorded. Follow-up data were collected for all patients up to the end of June 2025.

Continuous variables were summarized as median [interquartile range, IQR] and compared between groups using the Mann–Whitney U test. Categorical variables were expressed as counts and percentages; 2 × 2 tables were analyzed with Fisher’s exact test, while larger contingency tables were assessed using the Fisher–Freeman–Halton exact test or its Monte Carlo approximation. Ordinal variables (e.g., gravidity, parity, and pregnancy outcome) were compared using the Cochran–Armitage trend test. All tests were two-sided, with a significance level of *p* < 0.05. PFS was defined as the time from primary surgery to the first recurrence or last follow-up without progression. Since COS was performed after FSS and at variable times during follow-up, analyses accounted for the potential immortal time bias, i.e., the period during which recurrence cannot occur before COS.

The primary analysis used a time-dependent Cox proportional hazards model, in which COS was modeled as a time-varying exposure switching from 0 (not yet stimulated) to 1 (stimulated) at the time of oocyte pick-up. This approach allows for appropriate alignment of exposure with time at risk. As a sensitivity analysis, a landmark analysis was performed, with the landmark time set at the median time from surgery to COS in the study population. Only patients who were recurrence-free at that time were included, and exposure status (“stimulated before the landmark” vs “not stimulated”) was defined accordingly. PFS after landmark was analyzed using a Cox model. The proportional hazards assumption was verified using Schoenfeld residuals. Given the limited number of events observed in the cohort, multivariable Cox regression models were deliberately not performed in order to avoid model overfitting and unstable estimates. The primary analytical focus was therefore placed on correct temporal alignment of exposure, using time-dependent and landmark approaches to address immortal time bias. Residual confounding related to baseline clinical differences between stimulated and non-stimulated patients cannot be fully excluded and is acknowledged as an inherent limitation of this exploratory analysis. Analyses were performed using R software (version 4.3.1) with the survival and survminer packages.

The study protocol (ID 7607) was approved by the local ethics committee (Lazio Area 3). All patients included in the present analysis gave written consent that covered the collection and use of personal health data for research purposes, in compliance with ethical standards and in the absence of any identifiers linking individuals to the dataset.

## 3. Results

Fifty-six patients diagnosed with BOT were screened. Seven patients (12.5%) were excluded because they underwent COS and OC after a recurrence of BOT, three patients (5.4%) were excluded owing to stromal microinvasion greater than 5 mm^2^, and one patient was lost to follow-up (1.8%). Ultimately, 45 patients with BOT who underwent FSS at first diagnosis were included in the analysis. Among them, 19/45 (42.2%) underwent COS and OC following FSS while the remaining 26/45 patients (57.8%) did not undergo COS after FSS.

The patients’ age at diagnosis ranged from 23 to 32.5 years, with a median age of 28 years. Patient-related characteristics are shown in Table 1. No statistically significant differences were observed between groups for age, BMI, smoking status, or family status.

BOT-related characteristics are summarized in Table 2.

Overall, bilateral disease was observed in 16 patients (35.6%), and the most frequent histological subtype was serous. Different FSS approaches were performed, including cystectomy, unilateral salpingo-oophorectomy, and unilateral salpingo-oophorectomy with contralateral cystectomy. While no statistically significant differences were detected between groups regarding tumor laterality, type of fertility-sparing surgery, implant status, International Federation of Gynecology Oncology (FIGO) stage, or histological subtype, the no-COS group tended to include a higher proportion of patients with bilateral disease and more extensive conservative surgical procedures.

The median duration of follow-up (FU), estimated by the reverse Kaplan–Meier method, was 34.4 months (95% CI, 22.6–46.2), with no relevant differences between groups (no-COS group: 31.8 months, 95% CI 24.9–38.6; COS-OC group: 35.1 months, 95% CI 6.8–63.3). No disease-related deaths occurred during FU. Overall, 14 patients (31.1%) experienced at least one recurrence.

An exploratory stratified analysis according to surgical procedure (cystectomy vs other fertility-sparing procedures) was performed. Recurrence rates were similar between groups (36.8% vs 26.9%; Fisher’s exact *p* = 0.525), as were pregnancy rates (21.1% vs 26.9%; Fisher’s exact *p* = 0.736).

The crude recurrence rate in the COS-OS group was 21.1% (4/19) and 38.5% (10/26) in the no-COS group (*p* = 0.330). These unadjusted comparisons are reported for descriptive purpose only and do not account for the delayed initiation of COS during follow-up.

**Figure 1:**
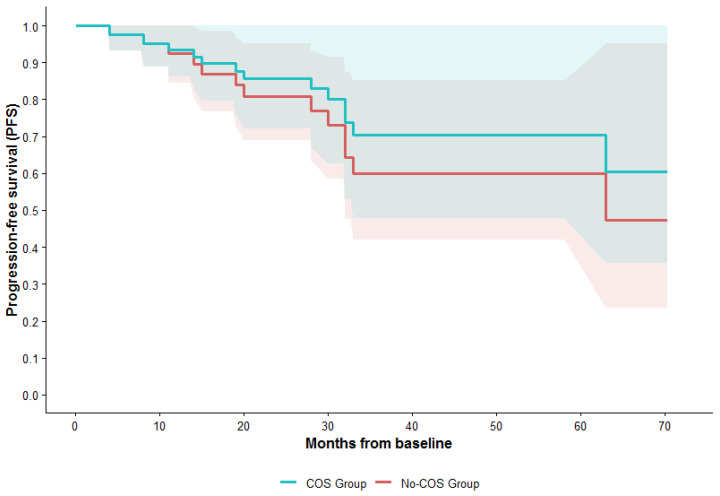
Predicted progression-free survival from the time-dependent Cox model (COS as a time-varying covariate). Model-based predicted progression-free survival (PFS) from a time-dependent Cox model, where controlled ovarian stimulation (COS) is treated as a time-varying covariate. Patients are considered “not stimulated” until the date of oocyte pick-up, and “stimulated” thereafter. The red curve represents patients who were never stimulated during follow-up, while the blue curve shows survival predicted after stimulation. Shaded areas indicate 95% confidence intervals.

After accounting for immortal time bias, no evidence of an association between COS and recurrence risk was observed. In the time-dependent Cox proportional hazards model, COS was not associated with PFS (HR 0.95; 95% CI 0.22–4.09; *p* = 0.994). Consistent findings were observed in the landmark analysis, in which the hazard ratio for recurrence was 0.37 (95% CI < 0.01–5.78, *p* = 0.523). Both analytical approaches yielded concordant results, showing no detectable signal of increased recurrence risk associated with COS. However, the wide confidence interval reflects the limited number of events and variability in the timing of COS initiation and follow-up duration, and precludes a definitive conclusion regarding a causal relationship between COS and oncologic outcomes (Figure 1).

Among the 19 patients in the COS–OC group, seven (36.8%) had previously undergone cystectomy. Given that oocyte retrieval may involve transvaginal puncture of residual ovarian tissue on the previously affected side, careful pre-procedural assessment was performed to ensure oncologic safety. This evaluation included physical examination, transvaginal ultrasound, and serum CA-125 measurement to exclude the presence of residual disease.

According to patient-reported information, as COS and OS were performed outside our center, when cystectomy had been carried out and residual ovarian tissue exhibited accessible follicles, oocyte retrieval was performed bilaterally in order to maximize the number of oocytes collected.

All recurrences were ovarian and were confirmed by histopathological evaluation. In the COS-OS group, three women who experienced recurrence underwent cystectomy; notably, none of these recurrences had peritoneal invasive implants. One woman was awaiting surgery at the time of the study. In the no-COS group, 10 patients had at least one recurrence, with 3/10 (30.0%) experiencing two recurrences and one patient experiencing three. Among the recurrences in the no-COS group, five were bilateral. In the no-COS group with implants, one case was invasive, and two were non-invasive. Two patients underwent radical surgery: one patient after her second recurrence and the other after her third recurrence.

Before BOT diagnosis, two women had a live birth, one by cesarean section and the other by vaginal delivery, whereas all remaining patients were nulligravid and nulliparous. All fertility outcomes reported below refer to pregnancies occurring after BOT diagnosis and FSS. After FSS, all women self-reported their desire to conceive during clinical consultation. During follow-up, 11 patients (24.4%) achieved at least one pregnancy, seven of which had one live birth each, and the remaining four had a total of seven miscarriages. Pregnancy rates did not differ significantly between patients who underwent COS and OC, and those who did not (21.1% vs 26.9%, respectively; *p* = 0.736). Fertility outcomes are detailed in Table 3.

Most pregnancies were achieved through unassisted conception (9/11, 81.8%). In the COS-OC group, three out four women who became pregnant conceived spontaneously. Assisted conception through in vitro fertilization (IVF) thawing cryopreserved oocyte was observed in one patient, accounting for three pregnancies, all of which resulted in miscarriage. In the no-COS group, three women experienced a total of four pregnancies, all ending in miscarriage, and one pregnancy was ongoing at the time of last follow-up. One patient of the no-COS group had a live birth through oocyte donation.

None of the patients who achieved a pregnancy experienced BOT recurrence after pregnancy. As recommended in the ESGO-ESHRE-ESGE guidelines [4], no routine completion surgery (removal of remaining ovary and tube) was performed (level of evidence IV, grade D).

## 4. Discussion

The study provides exploratory evidence on oncologic and reproductive outcomes in patients with BOT undergoing FSS, with or without subsequent COS and OC. After accounting for delayed COS initiation and correcting for immortal time bias, no signal of increased recurrence risk associated with COS was identified. PFS and post-treatment fertility outcomes were also evaluated.

Interpretation of oncologic results in this setting requires particular caution, given the retrospective design, limited number of events, and non-randomized COS exposure. Although crude comparisons suggested lower recurrence rates in the COS group, time-dependent and landmark analyses accounting for exposure timing showed no association between COS and recurrence risk. Importantly, the wide confidence intervals indicate substantial statistical uncertainty, supporting the conclusion that, within the constraints of the data, no evident increase in recurrence risk associated with COS was observed.

No differences in recurrence or pregnancy outcomes were observed according to type of fertility-sparing surgery in this exploratory analysis.

When considering the safety of COS and OC, it is important to acknowledge the procedural risks associated with oocyte retrieval. A potential mechanism by which oocyte retrieval could theoretically increase the risk of recurrence or peritoneal dissemination relates to the inadvertent release of tumor cells during follicular puncture. If residual borderline tissue persists in the operated or contralateral ovary, or if a new lesion has developed, disruption of the ovarian capsule may allow the dissemination of cells with borderline potential into the peritoneal cavity, with subsequent implantation on peritoneal surfaces. However, to date, no studies have specifically quantified this risk in the context of COS and oocyte retrieval performed on a previously operated ovary for BOT, and addressing this question remains inherently challenging due to the multifactorial nature of recurrence and the biological behavior of BOTs.

In this context, thorough pre-procedural evaluation and individualized risk assessment remain essential to ensure oncologic safety. However, despite this theoretical concern, the available evidence—including our findings—does not support a clinically significant increase in recurrence risk associated with COS and oocyte retrieval following FSS. Consistent results have been reported in the recent literature, including the study by Liang et al. [18], which found that COS could be safely conducted post-FSS without increasing the recurrence risk of BOT.

With regard to reproductive outcomes, the observed post-treatment pregnancy rate was 24.4%, which is lower than the rates reported in previous reviews, ranging from 32% to 88% [19]. This difference may be partly explained by the surgical characteristics of our cohort, as 57.8% of patients underwent unilateral salpingo-oophorectomy with or without contralateral cystectomy. Consistent with this, Palomba et al. reported significantly higher pregnancy rates after cystectomy compared with salpingo-oophorectomy-based procedures [20].

Within these limitations, our data provide descriptive evidence that pregnancy is achievable after FSS for BOT, frequently without the use of OC. In view of the limited percentage of assisted pregnancy (18.2%), the role of controlled ovarian stimulation and oocyte cryopreservation in this clinical setting should be thoughtfully and individually considered. As suggested by Cosyns et al., in patients with early-stage BOT treated conservatively, fertility preservation strategies may primarily alleviate psychological distress related to recurrence risk and potential decline in ovarian reserve due to repeated surgeries, rather than substantially increasing pregnancy rates [21].

Two additional ethical considerations deserve attention. First, the percentage of women who ultimately return to use cryopreserved oocytes is consistently low. In our cohort, one patient (1/19, 5.3%) thawed her oocytes, resulting in three miscarriages, in line with a previous report. Cosyns et al. similarly observed no utilization of cryopreserved oocyte among 13 BOT patients undergoing OC after FSS, despite eight achieving spontaneous pregnancies [21]. Extending our search to any oncological patients who underwent COS and OC, Zimmermann et al. [22] reported a 12-year experience of fertility preservation prior to oncological treatment in a French center. Three hundred and twenty-seven patients were included, and the return rate of cryopreserved oocytes was 6.9% (22/318). Another study conducted by Porcu et al. [23] analyzed 508 oncological patients, 83 of which had gynecological disease. During 25 years of fertility preservation, the study reported a return rate of cryopreserved oocytes of 8.7% (44/508).

The second aspect involves the social cost of COS. In the literature, the analyses about the cost-effectiveness of COS are exiguous. One study by Beresniak A et al. used the Italian Health System as a reference model to reflect a real-world scenario of the cost-effectiveness of COS [24]. From the National Health Service (NHS) perspective, the study reported a mean cost per successful pregnancy of € 13.574 ± 6157 when COS was carried out [24]. It is important to note that the Italian NHS entirely covers the costs of the gonadotropins used for COS, regardless of whether they are carried out in public or private clinics [25]. Considering all these aspects, physicians must carefully evaluate whether COS should be offered to all BOT patients, given its growing economic burden on the NHS.

Several limitations of this study should be acknowledged. First, the retrospective single-center design and the limited sample size restrict the statistical power to detect small differences in recurrence risk and preclude definitive causal inferences. Second, although the median follow-up duration was adequate for an exploratory analysis, the wide range of follow-up reflects heterogeneity in patient enrolment over time and may contribute to variability in outcome estimates. Furthermore, COS and OC were performed at non-affiliated centers, outside FPG; therefore, detailed information on ovarian stimulation protocols, number of cycles, and oocyte yield was not consistently available.

In addition, allocation to controlled ovarian stimulation was not randomized and may have been influenced by clinical and patient-related factors, resulting in baseline differences between groups that cannot be fully accounted for. For this reason, the present analysis focused on appropriate temporal handling of exposure rather than multivariable adjustment, which would have been unstable given the limited number of events.

Nevertheless, the study also has relevant strengths, including systematic collection of oncologic and reproductive outcomes, detailed characterization of surgical management, and the use of time-dependent and landmark approaches to address immortal time bias, a methodological issue rarely accounted for in previous studies on this topic.

In conclusion, in this exploratory monocentric cohort, COS after FSS in patients with BOTs did not show signals of increased BOT recurrence after correction for immortal time bias within a short- to mid-term follow-up. Reproductive outcomes were favorable, with most pregnancies occurring through unassisted conception. Nevertheless, larger, adequately powered studies are required to confirm these findings. To address this unmet need, a multicenter study protocol has been submitted to the MITO (Multicenter Italian Trials in Ovarian Cancer and Gynecologic Malignancies) group, designed to assess the safety and efficacy of COS following FSS in BOT patients across leading Italian centers. Looking ahead, we anticipate that stronger scientific evidence—evaluating not only efficacy and safety but also cost-effectiveness within the framework of the national health system (NHS)—will be instrumental in shaping ethical and public health guidelines, informing policy decisions, and supporting evidence-based clinical recommendations.

## Figures and Tables

**Table 1:** Patient-related characteristics.

Characteristics	Overall *n* = 45	COS-OC Group *n* =19 (42.2%)	No-COS Group *n* = 26 (57.8%)	*p*-Value
Age in years	28.0 [23.0–32.5]	25.0 [23.0–32.0]	29.0 [25.8–33.0]	0.189
BMI in kg/m^2^	22.2 [20.3–25.1]	22.0 [20.2–23.2]	23.2 [20.1–27.9]	0.260
Smoke				0.569
No	32 (71.1)	12 (63.2)	20 (77.0)	
Yes	7 (15.6)	4 (21.1)	3 (11.5)	
Ex	6 (13.3)	3 (15.7)	3 (11.5)	
Family status				0.480
Single	11 (25.6)	6 (33.3)	5 (20.0)	
Partnered	32 (74.4)	12 (66.7)	20 (80.0)	

Results are presented as median (range) or *n* (%) as appropriate. Comparisons were made with two-sided Mann–Whitney U test for continuous variables, Fisher’s exact test or Fisher–Freeman–Halton test for categorical variables, and the Cochran–Armitage trend test for ordinal variables. BMI: Body Mass Index. COS: controlled ovarian stimulation; OS: oocyte cryopreservation.

**Table 2:** BOT-related characteristics.

Characteristics	Overall *n* = 45	COS-OC Group *n* = 19 (42.2%)	No-COS Group *n* = 26 (57.8%)	*p*-Value
Laterality				0.206
Right	17 (37.8)	8 (42.1)	9 (34.6)	
Left	12 (26.6)	7 (36.8)	5 (19.2)	
Bilateral	16 (35.6)	4 (21.1)	12 (46.2)	
Type of surgery				0.246
C	19 (42.2)	7 (36.8)	12 (46.2)	
USO	18 (40.0)	10 (52.7)	8 (30.7)	
USO + CC	8 (17.8)	2 (10.5)	6 (23.1)	
FIGO stage				n.a.
IA	3 (6.7)	1 (5.3)	2 (7.7)	
IB	4 (8.9)	1 (5.3)	3 (11.6)	
IC	2 (4.4)	1 (5.3)	1 (3.8)	
IIB	3 (6.7)	2 (10.4)	1 (3.8)	
III	1 (2.2)	-	1 (3.8)	
No formal staging	32 (71.1)	14 (73.7)	18 (69.3)	
Histology				0.727
Serous	28 (62.4)	11 (57.9)	17 (65.4)	
Mucinous	4 (8.8)	2 (10.5)	2 (7.7)	
Mixed	13 (28.8)	6 (31.6)	7 (26.9)	
Implants				0.33
No	33 (73.3)	14 (73.7)	19 (73.1)	
Not invasive	10 (22.2)	4 (21.0)	6 (23.1)	
Invasive	2 (4.5)	1 (5.3)	1 (3.8)	

Results are presented as median (range) or *n* (%) as appropriate. Comparisons were made with two-sided Mann–Whitney U test for continuous variables, Fisher’s exact test or Fisher–Freeman–Halton test for categorical variables, and the Cochran–Armitage trend test for ordinal variables. C: cystectomy; USO: unilateral salpingo-oophorectomy; USO + CC: unilateral salpingo-oophorectomy + contralateral cystectomy; FIGO: International Federation of Gynecology Oncology; n.a.: not assessable; BOT: Borderline Ovarian Tumour.

**Table 3:** Fertility outcomes after BOT.

Characteristics	Overall *n* = 45	COS-OC Group *n* = 19 (42.2%)	No-COS Group *n* = 26 (57.8%)	*p*-Value
Fertility outcomes				0.736
No pregnancy	34 (75.6)	15 (78.9)	19 (73.1)	
Yes pregnancy	11 (24.4)	4 (21.1)	7 (26.9)	
Conception				>0.9
Spontaneous	9 (81.8)	3 (75.0)	6 (85.7)	
IVF	2 (18.2)	1 (25.0)	1 (14.3)	
Pregnancy outcomes				>0.9
Miscarriage	4 (36.4)	1 (25.0)	3 (42.9)	
Live birth	7 (63.6)	3 (75.0)	4 (57.1)	
Miscarriage				n.a.
1 M/C	2 (4.4)	0	2 (7.7)	
2 M/C	1 (2.2)	0	1 (3.8)	
3 M/C	1 (2.2)	1 (5.3)	0	

Results are presented as *n* (%). Comparisons were made with Fisher’s exact test or the Fisher–Freeman–Halton test for categorical variables, and the Cochran–Armitage trend test for ordinal variables. IVF: in vitro fertilization; M/C: Miscarriage. n.a.: not assessable.

## Data Availability

The data presented in this study are available on request from the corresponding author.
